# Heat Shock Protein Inspired Nanochaperones Restore Amyloid‐β Homeostasis for Preventative Therapy of Alzheimer's Disease

**DOI:** 10.1002/advs.201901844

**Published:** 2019-09-16

**Authors:** Huiru Yang, Xinyu Li, Lin Zhu, Xiaohui Wu, Shaozhi Zhang, Fan Huang, Xizeng Feng, Linqi Shi

**Affiliations:** ^1^ State Key Laboratory of Medicinal Chemical Biology Key Laboratory of Functional Polymer Materials Ministry of Education Institute of Polymer Chemistry College of Chemistry Nankai University Tianjin 300071 P. R. China; ^2^ State Key Laboratory of Medicinal Chemical Biology Key Laboratory of Bioactive Materials Ministry of Education College of Life Sciences Nankai University Tianjin 300071 P. R. China; ^3^ Tianjin Key Laboratory of Radiation Medicine and Molecular Nuclear Medicine Institute of Radiation Medicine Chinese Academy of Medical Sciences & Peking Union Medical College Tianjin 300192 P. R. China

**Keywords:** Alzheimer's disease, amyloid beta peptides, polymeric micelles, precaution, self‐assembly nanochaperones

## Abstract

Amyloid beta (Aβ) aggregation is generally believed as the crucial and primary cause of Alzheimer's disease (AD). However, current Aβ‐targeted therapeutic strategies show limited disease‐modifying efficacy due to the irreversible damages in the late stage of AD, thus the treatment should be given before the formation of deposition and target primary Aβ species rather than advanced plaques. Herein, inspired by heat shock protein, a self‐assembly nanochaperone based on mixed‐shell polymeric micelle (MSPM) is devised to act as a novel strategy for AD prevention. With unique surface hydrophobic domains, this nanochaperone can selectively capture Aβ peptides, effectively suppress Aβ aggregation, and remarkably reduce Aβ‐mediated cytotoxicity. Moreover, the formed nanochaperone‐Aβ complex after Aβ adsorption can be easily phagocytosed by microglia and thereby facilitates Aβ clearance. As a result, the nanochaperone reduces Aβ burden, attenuates Aβ‐induced inflammation, and eventually rescues the cognitive deficits of APP/PS1 transgenic AD mice. These results indicate that this biomimetic nanochaperone can successfully prevent the onset of AD symptoms and serve as a promising candidate for prophylactic treatment of AD.

## Graphical abstract

A heat shock protein inspired nanochaperone is devised for the preventative therapy of Alzheimer's disease (AD). With the excellent effects in inhibiting Aβ aggregation, mitigating Aβ‐mediated cytotoxicity, and facilitating Aβ clearance, this nanochaperone successfully rescues cognitive deficits and reduces Aβ burden of APP/PS1 transgenic AD mice, thus indicating that the nanochaperone can effectively suppress the progression of early AD.

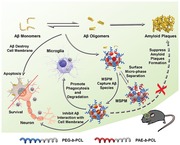

## 1 Introduction

Alzheimer's disease (AD) is the most prevalent and intractable age‐related progressive neurodegenerative disorder with a growing amount of patients and cost.^1^ Current available anti‐AD drugs can only alleviate the symptoms temporarily rather than cure this disease. The abundant extracellular senile plaques formed by amyloid beta peptides (Aβ) in cerebrum are considered as the crucial identified pathological hallmark of AD.^2^ Thus, researchers proposed amyloid cascade hypothesis which maintained that the accumulation of Aβ was the chief culprit in AD pathogenesis and led to many secondary pathological processes.^3^ Once the Aβ homeostasis was disrupted, the amount of Aβ would increase and form various toxic aggregates, which could lead to the Aβ‐mediated cytotoxicity and inflammation, ultimately resulting in serious neuron damage and cognitive impairment.^4^, ^5^ Based on this hypothesis dominating the AD research field, a great deal of efforts has been devoted to suppress Aβ aggregation and eliminate its deposition, mainly including small organic molecules,^6^ peptides,^7^ and Aβ‐specific antibodies.^8^ However, the disease‐modifying efficacy of these strategies was limited and came with serious adverse effects.^9^, ^10^ Some studies attributed these failures to the invalid intervention at the late stage of AD, in which the neurological damage was irreversible and difficult to be healed via simply eliminating Aβ plaques.^11^ Since the AD progression gradually became less dependent on Aβ in late stage,^12^ it has become a consensus that the Aβ‐targeting therapy should be carried out before the prevalence of plaques and overt symptoms. Thus, it would be more promising to develop a preclinical Aβ‐targeting therapy and convert the therapeutic target from intricate Aβ plaques to relatively simple monomers and oligomers, thereby preventing the occurrence of consequent symptoms.

Molecular chaperone is an important component of cellular quality control system, which maintains intricate proteostasis in life entity.^13^ Among various chaperones, heat shock proteins (HSPs) play a vital role in inhibiting protein misfolding and aggregation, assisting the across‐membrane transport of proteins and facilitating protein degradation.^14^, ^15^ Typically, with hydrophobic binding sites, HSPs could recognize and bind to surface‐exposed hydrophobic residues of aberrant proteins, thereby sequestrating abnormal proteins each other and preventing their unfavorable aggregation.^16^, ^17^ Furthermore, these chaperones not only protect neuron by avoiding the harmful contact of toxic proteins on the cell membrane,^18^, ^19^ but also accelerate the clearance of paraproteins by facilitating their transmembrane transport, phagocytosis, and degradation.^20^, ^21^, ^22^ As a typical protein aggregation neurodegenerative disease, AD is mainly caused by the excessive production and accumulation of aberrant Aβ peptide in the extracellular matrix. Although HSPs are important maintainers of Aβ homeostasis,^23^, ^24^ they primarily work in the intracellular cytoplasm.^25^, ^26^ Thus, introducing exogenous HSPs is an effective therapeutic strategy for AD. However, the difficulty in extraction and expensive price severely limited the clinical use of natural HSPs. Therefore, an alternative artificial nanochaperone with similar functions to HSPs would be an ideal candidate to fight against AD. In order to achieve this aim, the artificial nanochaperones should i) own hydrophobic recognize sites to capture Aβ peptides, ii) have appropriate barriers to isolate Aβ peptide, inhibiting their aggregation and blocking the harmful adhesion between Aβ and cell membrane, iii) resist interference from other proteins in complex biological environment, and iv) promote the transport of Aβ into microglia, facilitating Aβ phagocytosis and clearance.

**Scheme 1:**
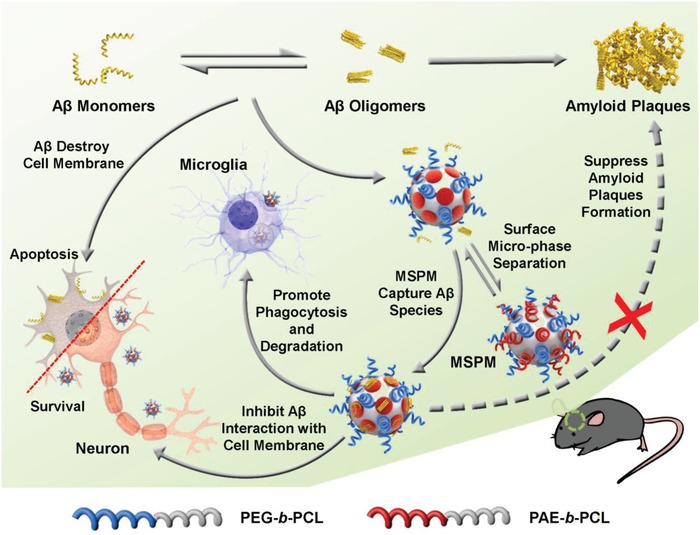
Illustration of the nanochaperone mechanism of action. By capturing Aβ species, nanochaperone suppresses Aβ aggregation and mitigates Aβ‐mediated cytotoxicity, as well as facilitates microglia phagocytosis of Aβ, thus protecting nerve cells against apoptosis and promoting the restoration of Aβ homeostasis in vivo.

Herein, inspired by natural HSPs, we constructed a biomimetic self‐assembly nanochaperone based on the biodegradable mixed‐shell polymeric micelle (MSPM) to serve as a novel prophylactic therapy for AD (**Scheme** 1). This nanochaperone is very suitable for in vivo application due to its favorable biocompatibility and biodegradability of all segments in polymers.^27^ The efficacy of nanochaperone is mainly derived from the phase separation structure composed of the surface hydrophobic microdomains and hydrophilic chain segments. In the physiological conditions, the hydrophobic surface microdomains recognized and bound to Aβ, while hydrophilic segments acted as barriers to separate Aβ from each other, thus allowing the nanochaperone to capture Aβ peptide and subsequently inhibit Aβ aggregation. With the microphase separation structure, the MSPM‐based nanochaperone showed high affinity for Aβ and was able to capture Aβ selectively even under the interference of other proteins in complex biological milieu. Similar with natural HSPs, the hydrophobic recognition sites and separation chamber endowed the nanochaperone with excellent ability to reduce Aβ adhesion to cell membranes and protect neuron from Aβ‐mediated cytotoxicity. Moreover, after the adsorption of Aβ, the formed nanochaperone‐Aβ complex was susceptible to be endocytosed by microglia and thereby facilitated Aβ clearance. Furthermore, we found that our nanochaperone prominently reduced Aβ burden, attenuated Aβ‐induced inflammation, and rescued the cognitive deficits of APP/PS1 transgenic AD model mice at the early stage of disease. Therefore, this MSPM‐based nanochaperone would be a promising tactic for early AD precaution.

## 2 Results and Discussion

### 2.1 Preparation and Characterization of Nanochaperone

In this study, MSPM‐based nanochaperone was fabricated by straightforward self‐assembly of poly(β‐amino ester)‐*block*‐poly(ε‐caprolactone) (PAE‐*b*‐PCL) and poly(ethylene oxide)‐*block*‐poly(ε‐caprolactone) (PEG‐*b*‐PCL) with three different weight ratios (2:1, 1:1, and 1:2), and the single PEGylated micelle (PM) based on merely PEG‐*b*‐PCL was acted as control. The synthetic route of two diblock copolymers and their chemical composition characterization of ^1^H NMR are shown in Figures S1 and S2 (Supporting Information), respectively. The nanochaperone was assembled in weak acid aqueous solution, forming a complex micelle with a hydrophobic PCL core and mixed shell comprised of PAE and PEG. After dialysis in a phosphate buffer solution (PBS, 10 × 10^−3^ m, pH 7.4), the surface PAE chains would turn into hydrophobic state and collapse on the core due to deprotonation, thus forming hydrophobic domains and adaptive surface cavities between outstretched PEG segments. The size distribution and morphology of these micelles were characterized by dynamic light scattering (DLS) and transmission electron microscopy (TEM), and their surface charges were evaluated by zeta potential measurements. As shown in Figure S3 (Supporting Information), the prepared MSPMs were all spherical with similar average sizes around 88 nm, while the PM had a diameter of about 75 nm that was smaller than MSPMs, probably due to the single composition of PM. The charge conversion phenomena of three MSPMs from pH 5.0 to pH 7.4 successfully confirmed the collapse of PAE chains and thus formation of hydrophobic domains at normal physiological conditions (Figure S4, Supporting Information).

### 2.2 Nanochaperone Inhibits Aβ Aggregation In Vitro

**Figure 1:**
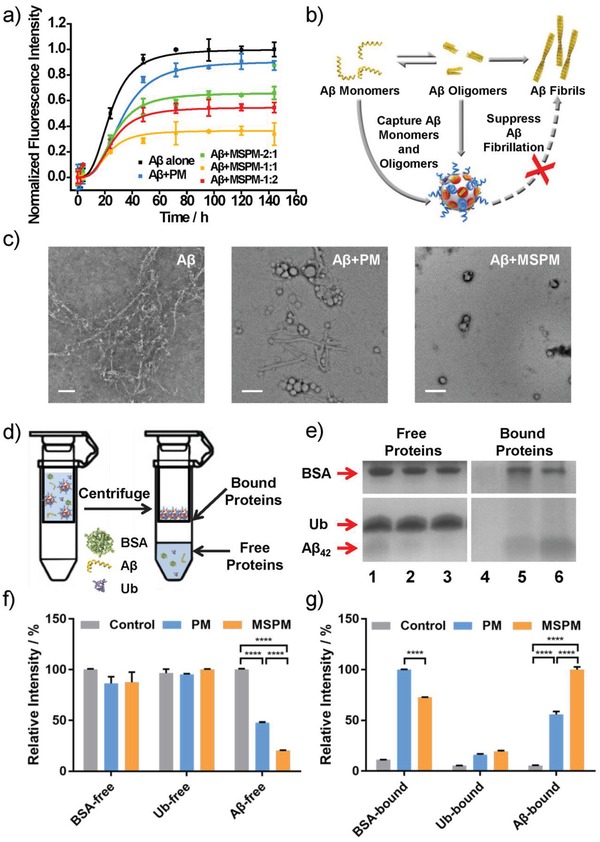
In vitro inhibition effects of nanochaperone on Aβ aggregation and its antiprotein interference ability. a) Fibrillation kinetics of Aβ at 37 °C in the absence or presence of MSPMs with different hydrophilic/hydrophobic ratios. Data were presented as mean ± SD, *n* = 3. b) Illustration of inhibiting Aβ aggregation by nanochaperones. c) TEM images of Aβ incubated with or without micelles at 37 °C for 5 d. The weight ratio of MSPM was 1:1(w/w). Scale bar = 200 nm. d) Schematic representation of the separation of free proteins and bound proteins to investigate the Aβ‐binding ability of nanochaperone. e) SDS‐PAGE analysis of the amount of three kinds of proteins (Aβ, BSA and ubiquitin) treated with or without micelles in the protein mixture. Proteins that were not bound to the micelles (left: free proteins) were separated from those micelle‐bound proteins (right: bound proteins). Lanes 1 and 4: no micelles; Lanes 2 and 5: treated with PM; Lanes 3 and 6: treated with MSPM. Quantitative analysis of protein content in f) free proteins and g) bound proteins by gray scale analysis of the band in (e). The relative intensity is the ratio of the intensity of each band to the strongest band in its group. The detail of data processing is given in the “Experimental Section.” Data were presented as mean ± SD, *n* = 3. One‐way ANOVA, ****p < 0.0001.

To testify the inhibitory effect of these nanochaperones on Aβ aggregation, we employed the thioflavin‐T (ThT) fluorescence assay to monitor the formation of amyloid aggregates. It is well known that ThT dye molecule can specially bind to Aβ species and its fluorescence intensity will increase with the evolution of Aβ aggregation.^28^ As seen in **Figure** 1a, when Aβ was incubated alone at 37 °C, the change of ThT fluorescence intensity displayed a sigmoidal shape, indicating that the formation of Aβ aggregates is a nucleation dependent polymerization process.^29^ Upon the addition of MSPMs, the ThT fluorescence intensity significantly decreased, which demonstrated that MSPMs possessed suppressing ability for Aβ aggregation. In contrast, PM only induced a little decrease of fluorescence intensity, highlighting the importance of hydrophobic domains in preventing Aβ aggregation. Moreover, compared with MSPM‐2:1 and MSPM‐1:2, MSPM‐1:1 exerted superior inhibiting effect, which suggested that the balance of surface hydrophilic/hydrophobic was a key factor for improving their performance. Thus, we chose MSPM‐1:1 for the subsequent experiments and refer to it as MSPM.

To further verify the inhibition activity of nanochaperone in Aβ aggregate formation, TEM measurements were utilized to study the morphology changes of Aβ incubated with or without micelles. After 37 °C incubation for 5 d and stained with phosphotungstic acid, obvious large aggregates and long fibrils were observed in Aβ alone sample and short fibers were found in Aβ/PM mixture, respectively (Figure 1c). In contrast, Aβ was absorbed on the surface of MSPM and there were not any fibrous aggregates in the mixture of Aβ and MSPM. These results further supported above ThT data and indicated that MSPM could effectively inhibit Aβ aggregation.

### 2.3 Antiprotein Interference Ability and Aβ Binding Affinity of Nanochaperone

One of the biggest challenges for clinical application of Aβ inhibitors is the complicated biological environment in vivo. Typically, there are massive different protein species in biological fluid and they can interfere with the functionality of Aβ inhibitors. Thus, resisting these interferences is of great importance for any Aβ inhibitors while there were few reports about it. To evaluate the antiprotein interference ability of nanochaperone, the Aβ‐binding affinity of nanochaperone in protein mixture was assessed. Considering the abundance and the sizes of proteins, two widespread proteins in organisms, bovine serum protein (BSA, *M*_n_ = 66 kDa) and ubiquitin (Ub, *M*_n_ = 8.5 kDa), were chosen as models of interfering proteins. Aβ was first mixed with BSA and ubiquitin evenly and then incubated with MSPM and PM respectively. Sample without micelles was performed as control. The mixtures were separated by ultrafiltration as the illustration in Figure 1d. The protein contents in filtrate (Figure 1e, free proteins) and interception liquid (Figure 1e, bound proteins) were analyzed by sodium dodecyl sulfate‐polyacrylamide gel electrophoresis (SDS‐PAGE) assay. As shown in Figure 1e, there was no bound protein band in the gel of interception liquid of control group (Lane 4), indicating that all proteins could penetrate the ultrafiltration membrane and enter filtrate in the absence of micelles. However, after incubated with MSPM, the free Aβ band became much weaker while bound Aβ band got stronger (Figure 1e, Lanes 3 and 6). Moreover, the PM group displayed similar ability but the changes of Aβ band were not as obvious as MSPM group (Figure 1e, Lanes 2 and 5). These results suggested that MSPM was able to capture Aβ peptide in complex protein environment with a better capture capability than that of PM. In addition, for PM group, the weaker free BSA band and stronger bound BSA band showed that BSA could combine with PM to a certain extent, while MSPM exhibited weaker adsorption of BSA compared with PM, which benefited from the microphase separation structure on the surface of MSPM.^30^, ^31^, ^32^ As for ubiquitin, no obvious bands of ubiquitin were observed in bound protein gel, which demonstrated that there was almost no interaction between ubiquitin and micelles, no matter MSPM or PM. Quantitative analysis was shown in Figure 1f (free proteins) and 1 g (bound proteins), which further supported above observations. All these observations indicated that MSPM could capture Aβ peptide under the interference of other proteins, providing a great possibility for clinical application in vivo.

To further study the binding affinity of MSPM for Aβ and interfering proteins, the quartz crystal microbalance with dissipation monitoring (QCM‐D) measurement was exploited.^33^, ^34^ As shown in Figure S5 (Supporting Information), when MSPM was exposed to Aβ or other interfering proteins (BSA and ubiquitin), the resonant frequency decreased to varying degrees and the amount of frequency attenuation (△*f*) of Aβ was much larger than that of other proteins. This result indicated that the affinity of MSPM for Aβ was stronger than BSA and ubiquitin, thus endowing MSPM certain Aβ selectivity in complex surroundings, which was in accordance with the SDS‐PAGE results above.

The varied affinities of MSPM for different proteins can be attributed to the different interaction between micelles and proteins. For Aβ, its punchy affinity with MSPM primarily derives from the multitudinous noncovalent interactions with hydrophilic PEG chains,^35^ and the hydrophobic effect with hydrophobic PAE domains.^36^ The powerful multiple mutual effects offered MSPM a strong affinity to bind Aβ, resulting in the admirable inhibition effect on Aβ aggregation.^37^, ^38^ In contrast, due to the hydrophilic surface of BSA, the binding of BSA and MSPM only dependent on its interaction with PEG segments,^39^ thus weaker than Aβ. In the case of ubiquitin, its innate characters (rigid structure, high solubility, and strong stabilization)^40^ and the hydration layer of micelles provide little interaction between ubiquitin and MSPM, leading to the negligible affinity between them. In brief, MSPM offers varying interactions with diverse proteins, which is conducive to conquering the interference from massive different proteins in vivo.

### 2.4 Nanochaperone Mitigates Aβ‐Mediated Cytotoxicity and Protects Neuron In Vitro

**Figure 2:**
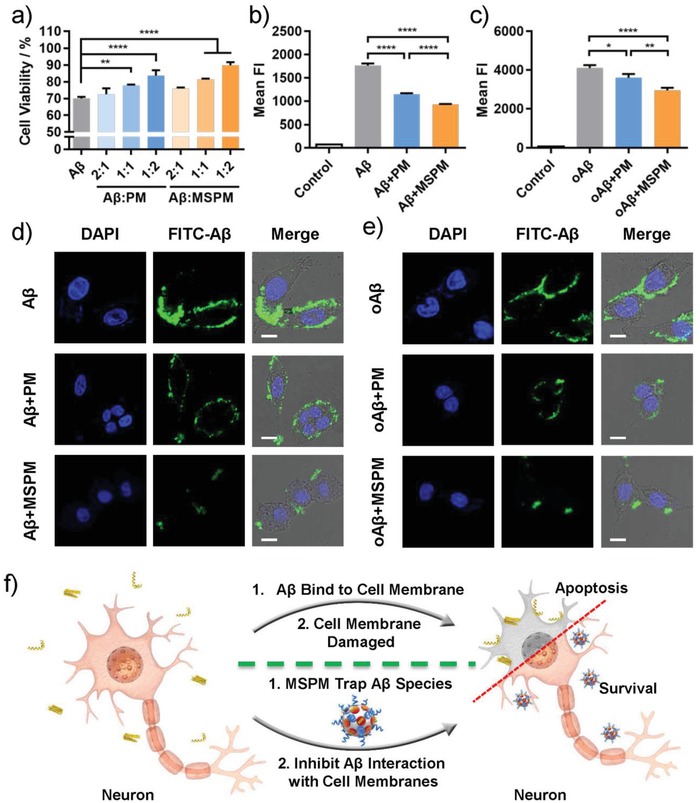
Reduction of Aβ‐mediated cytotoxicity by nanochaperone. a) Cell viabilities of PC‐12 cells after incubated with Aβ in the absence or presence of nanochaperone. Cell viability was measured by CCK‐8 assay. The flow cytometric quantification of the changes of the amount of Aβ b) monomer and c) oligomer interacting with PC‐12 cells in the absence or presence of micelles. Mean FI (Mean Fluorescence Intensity). All data were presented as mean ± SD, *n* = 3. One‐way ANOVA, *p < 0.05, **p < 0.01, and ****p < 0.0001. CLSM microscopy images of PC‐12 cells after incubation with FITC‐Aβ d) monomer or e) oligomer in the absence or presence of micelles. Scale bar = 10 µm. f) Illustration of nanochaperone inhibiting the interaction between Aβ species and cell membranes.

To evaluate the inhibition effects of MSPM on Aβ‐mediated cytotoxicity, PC‐12 cells were used as the neuron model and CCK‐8 assay was performed to measure the cell viability.^41^, ^42^ As shown in Figure S6 (Supporting Information), micelles with varying concentration from 25 to 400 µg mL^−1^ showed negligible cytotoxicity on PC‐12 cells with almost 100% cell viabilities, indicating the good biocompatibility of nanochaperone. When PC‐12 cells were treated with Aβ for 24 h, the cell viability was reduced to around 70% (**Figure** 2a). In the presence of MSPM, the survival rate of cells increased to 90% at most and this behavior showed a dose‐dependent manner, which demonstrated that nanochaperone could effectively reduce Aβ‐mediated cytotoxicity and protect neurons.

On the basis of the above results, we further investigated the protection mechanism of nanochaperone for nerve cells. Increasing evidence suggested that Aβ toxicity was directly related to their interaction with cell membranes, which led to membrane disruption and cell damage.^4^, ^5^ Moreover, it has been demonstrated that ATP‐independent molecular chaperones could inhibit the interaction between Aβ species and cell membranes.^43^ Thus, we aimed to survey whether our nanochaperone could mitigate Aβ‐mediated cytotoxicity though a similar mechanism. FITC labeled Aβ solution and micelles were added to PC‐12 cells in sequence, and the amount of Aβ interacting with cells was measured by confocal laser scanning microscopy (CLSM) and flow cytometry. As shown in Figure 2d,e, the Aβ alone group displayed obvious green fluorescence especially on cell surface, implying that Aβ monomers and oligomers were strongly bond with cell membranes. Nevertheless, the fluorescence intensity markedly decreased when introduced MSPM, indicating that MSPM could mitigate the adhesion of Aβ to cell surface and reduce the interaction of Aβ with cell membrane (Figure 2f). This inhibition of adhesion was attributed to the capture of Aβ species by the MSPM. Furthermore, it was noteworthy that PM also was able to prevent this interaction, but the effect was less than MSPM, perhaps because that they were not able to hide Aβ species well without surface cavity structures. The results of flow cytometry were in agreement with CLSM data (Figure 2b,c). Therefore, these results indicated that the protection effect of nanochaperone for neurons was due to the blocking of harmful interaction between Aβ and cell membranes.

### 2.5 Nanochaperone Facilitates Microglia Phagocytosis of Aβ

**Figure 3:**
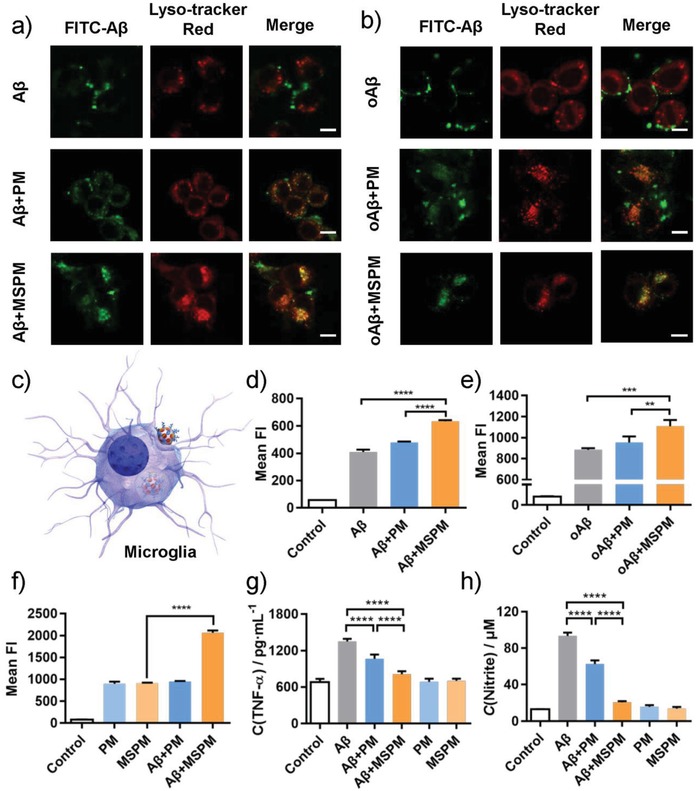
Facilitation effect of nanochaperone on microglia phagocytosis of Aβ and its attenuation effect on Aβ‐induced inflammation in vitro. CLSM microscopy images of BV‐2 cells after incubation with FITC‐Aβ a) monomer or b) oligomer in the absence or presence of micelles. Scale bar = 10 µm. c) Schematic illustration of the endocytosis of microglia for the complex of nanochaperone and Aβ. The flow cytometric quantification of internalized FITC‐Aβ d) monomer and e) oligomer by BV‐2 cells treated with or without micelles. Data were presented as mean ± SD, *n* = 3. f) The flow cytometric quantification of internalized PM‐Cy3 and MSPM‐Cy3 by BV‐2 cells treated with or without Aβ. Data were presented as mean ± SD, *n* = 3. g,h) The expression of pro‐inflammatory cytokines induced by Aβ in the absence or presence of micelles. g) TNF‐α; h) NO. Data were presented as mean ± SD, *n* = 4. One‐way ANOVA, **p < 0.01, ***p < 0.001, and ****p < 0.0001.

Microglia is the mononuclear phagocyte in the brain, which plays a crucial role in cerebral Aβ clearance.^44^ It could promote Aβ clearance by phagocytosis and depress the formation of Aβ deposition, thereby contributing to maintain Aβ homeostasis and delay AD disease progression in the early stage of AD. To examine the effect of nanochaperone on microglia‐mediated Aβ clearance, CLSM and flow cytometry were applied and murine microglial (BV‐2) cells were used as the model of microglia cells. As shown in **Figure** 3a,b, Aβ‐FITC treated cells exhibited little green fluorescence signal inside the cells, while MSPM treated group could markedly induce the internalization of Aβ species into cells, suggesting that MSPM‐Aβ complex was much easier to be uptake by microglia, and MSPM could significantly promote Aβ phagocytosis. Flow cytometry data (Figure 3d,e) also supported above results. Moreover, the colocalization of green Aβ‐FITC fluorescence and red lysosome fluorescence further confirmed that MSPM facilitated Aβ clearance through lysosomal network of microglia.

### 2.6 Cell Uptake of Nanochaperone and Nanochaperone‐Aβ Complex

In order to further investigate the reason of the nanochaperone reducing Aβ‐cell adhesion and promoting Aβ clearance, we studied the phagocytosis behavior of nanochaperone and nanochaperone‐Aβ complex by microglia. We labeled the micelles with Cy3 and evaluated the amount of internalized micelles by measuring the fluorescence intensity through flow cytometry. PM and MSPM with the same concentration had the same fluorescence intensity (Figure S7, Supporting Information). The results of CLSM showed that both nanochaperone and the complex could be phagocytized by microglia (Figure S8, Supporting Information). Without Aβ peptides, the similar fluorescence intensity of MSPM and PM in the results of flow cytometry mean their phagocytosis by microglia was analogous to each other (Figure 3f). However, after combined with Aβ, there was no obvious change for the phagocytosis level of PM‐Aβ complex compared with PM, while the MSPM‐Aβ complex showed much higher phagocytosis level than MSPM. These results indicated that micelle alone was less easy to be phagocytized by microglia than MSPM‐Aβ complexes, and inclined to stay in intercellular space, which is conducive to trap free Aβ peptides, inhibit their aggregation and decrease their interaction with cells, thus alleviating Aβ‐mediated neurotoxicity. Once the MSPM‐Aβ complexes formed, it would be easily to be phagocytized by microglia and promote the elimination of Aβ, which was consistent with the literatures.^45^ While for the low phagocytosis level of PM‐Aβ complex, we attributed this phenomenon to the weak Aβ‐binding ability of PM.

### 2.7 Nanochaperone Attenuates Aβ‐Induced Inflammation In Vitro

Extensive researches reported that Aβ could trigger the secretion of pro‐inflammatory cytokines, such as tumor necrosis factor (TNF‐α) and nitric oxide (NO), which would in turn damage microglia ability of Aβ phagocytosis and clearance. Thus, we next detected the effect of nanochaperone on Aβ‐stimulated cytokines release. Compared with control, Aβ treated cells displayed obvious increases of TNF‐α level and NO level (Figure 3g,h). In the presence of micelles, the levels of these two cytokines were largely reduced and this effect of MSPM group showed more pronounced. Meanwhile, coincubation with micelles alone had no influence on the level of TNF‐α and NO. These results demonstrated that MSPM could effectively decrease the production of proinflammatory cytokines and attenuate Aβ‐induced inflammatory.

### 2.8 The Retention and Distribution of Nanochaperones in Mice Brain

In view of the wonderful effects of nanochaperones in vitro, we hope to further verify its therapeutic effect on AD mice. Before the in vivo experiments, it is important to investigate the stability of nanochaperones in biological environment and their retention in mice brain first. The size changes of nanochaperones in fetal bovine serum (FBS) at 37 °C were monitoring though DLS (Figure S9, Supporting Information). The size distribution of the nanochaperones changed little after incubation at 37 °C for 25 d, and no aggregates were observed. Thus, the nanochaperones showed excellent stability in biological environment and were suitable for in vivo application.

To evaluate the retention and distribution of nanochaperones, the metabolic situation of micelles in mouse brain was first measured by injecting the TPETPAFN (a near‐infrared fluorescent probe)^46^ loaded or Cy3 labeled micelles in to mouse brain and monitoring by fluorescence imaging. Satisfactorily, the fluorescence retained in mouse brain over 25 d (Figure S10a,b, Supporting Information), and spread from the injection site into the bilateral cerebral cortex, hippocampus and parenchyma within 48 h (Figure S10b,c, Supporting Information), suggesting that one single injection of nanochaperone could diffuse into the entire brain with sustained effect for nearly a month.

The amount of MSPM in the bilateral cortex and hippocampus were quantified to further study the distribution of nanoparticles by monitoring the fluorescence intensity of Cy3‐MSPM in the brain. After injection, the micelle content in the right cortex and hippocampus decreased over time. Meanwhile, the micelle content in left cortex and hippocampus first increased and reached the maximum at day 2, indicating that nanochaperones could diffuse throughout the brain. Then, its content decreased with time because of the physiological metabolism. Furthermore, as time elapsed, the difference of micelle content between the right and left cortex or hippocampus diminished gradually, and the micelle content in bilateral cortex or hippocampus became almost equal from day 14 (Figure S10,e, Supporting Information). Considering the rapid and widespread distribution and long retention time of nanochaperones, it is reasonable to believe that nanochaperones could inhibit Aβ aggregation at an early age and prevent the onset of AD.

### 2.9 Nanochaperone Prevents Cognitive Deficits of APP/PS1 Transgenic Mice

**Figure 4:**
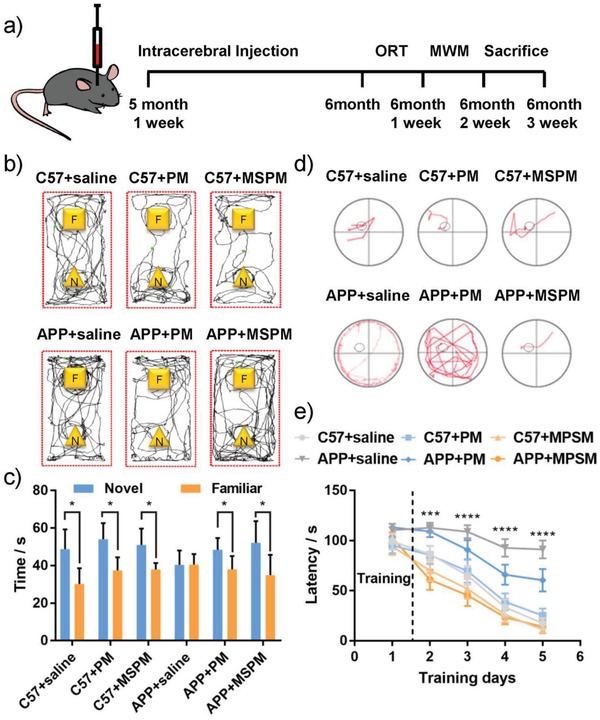
The improvement in memory performance of APP/PS1 mice. a) Time schedule of the experimental procedure. b) Representative tracing graphs and c) object exploring time in object recognition task (ORT) during the retention memory phase. Data were presented as mean ± SD, Student's *t*‐tests. Representative d) searching paths and e) escape latencies in Morris water maze (MWM) test. Data were presented as mean ± SD, Two‐way ANOVA. Four mice in APP/saline group and six mice in each other groups. C57: C57BL/6J mice; APP: APP/PS1 mice. Symbols (*) mark statistically significant difference between APP/saline group and APP/MSPM group. *p < 0.05, **p < 0.01, ***p < 0.001, and ****p < 0.0001.

Based on the capability of nanochaperone on Aβ aggregation and clearance, we then studied its preventive effect on cognitive deficits in APPswe/PS1ΔE9 (APP/PS1) transgenic mice. C57BL/6J (C57) mice were used as the wild‐type control (WT). According to the workflow in **Figure** 4a, saline, PM and MSPM were intracerebrally injected into the five‐month‐old mice before the Aβ plaques occurred. After intracerebral injection operation and recovery for 3 weeks, object recognition task (ORT) and Morris water maze (MWM) test were carried out to estimate the recognition and spatial memory of mice. In ORT test, three C57 mice groups all spent more time on exploring the novel objects in the test phase trials, while APP/saline group failed to discriminate familiar (F) and novel (N) objects (Figure 4b,c). In contrast, MSPM‐treated AD mice showed obvious preference for novel objects as WT controls, indicating that MSPM could markedly improve the recognition memory function of AD mice.

Meanwhile, in Morris water maze experiment, AD mice treated with MSPM showed a much shorter escape latency (Figure 4d,e) and swimming distance (Figure S11, Supporting Information) than APP/saline group, which was almost comparable to WT mice, suggesting that MSPM could significantly enhance the spatial localization ability and prevent the deterioration of spatial memory in AD mice. All above results of behavioral experiments demonstrated that nanochaperone can prevent cognitive deficits of APP/PS1 mice in the early stage of AD.

### 2.10 Nanochaperone Reduces Aβ Burden of APP/PS1 Transgenic Mice

**Figure 5:**
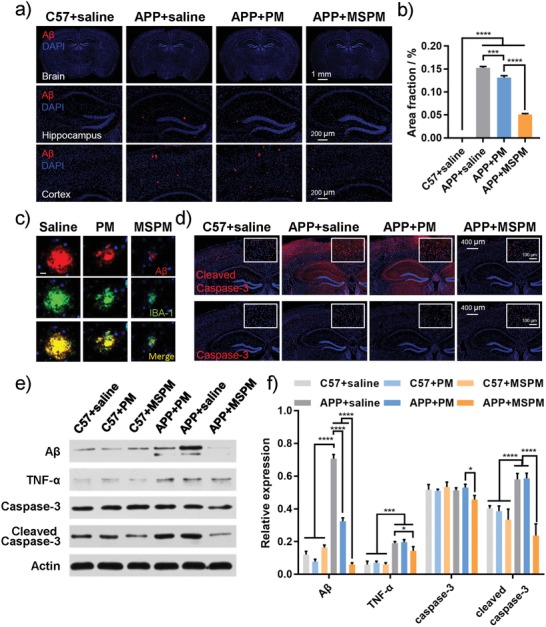
The reduction of Aβ burden in APP/PS1 mice. a) Immunofluorescence images for Aβ expression. Whole brain, hippocampus, and cortex were shown. Aβ: red; DAPI: blue. b) The plaque area ratio in mouse brain. c) Colocalization of Aβ (red) and IBA‐1 (green) in the brain of APP/PS1 mice and the typical representatives of the size changes of Aβ plaques. Bar = 10 µm. d) Immunofluorescence images for the expression of caspase‐3 and cleaved caspase‐3 in mice brain. e,f) Western blot analysis for whole brain and relative expression. All data were presented as mean ± SD, *n* = 3, One‐way ANOVA, *p < 0.05, **p < 0.01, ***p < 0.001, and ****p < 0.0001.

To explore whether our nanochaperone affected Aβ deposition in APP/PS1 mice brain, Aβ immunofluorescence staining was adopted to detect the quantity of amyloid plaques. As shown in **Figure** 5a, there were lots of amyloid plaques located in the cortex and hippocampus of APP/PS1 mice treated with saline, which was in sharp contrast to the C57 control mice. However, for the mice treated with MSPM, the amount and size of Aβ deposition were significantly decreased (Figure 5a–c, Figures S12 and S13, Supporting Information), indicating that MSPM administration was effective in reducing Aβ plaque expression in brain. Western blot data further proved above results (Figure 5e,f). Moreover, as a typical marker for microglia, IBA‐1 (green) was well colocalized with Aβ deposition (red), and the plaque areas in APP/PS1 mice were decreased when treated with micelles while MSPM treated group showed the smallest plaque (Figure 5c and Figure S12, Supporting Information). Furthermore, the immunofluorescence data also indicated that the nanochaperones could well colocalized with microglia in mouse brain (Figure S14, Supporting Information). This result further indicated that nanochaperones could promote the elimination of Aβ and depress the formation of Aβ plaques.

Considering the unilateral injection of nanochaperones, the Aβ levels in bilateral cerebral cortex and hippocampus were further compared. As shown in Figure S15 (Supporting Information), the result of ELISA assay exhibited no significant difference in Aβ levels between bilateral cerebral cortex or hippocampus in MSPM treated AD mice, which should be attributed to the wide distribution of nanochaperones in mouse brain.

Since the Aβ plaque burden was effectively relieved by MSPM administration, the question arose whether these micelles could be useful to suppress Aβ‐mediated inflammation and neuronal apoptosis in vivo. As shown in Figure 5d,f, the MSPM‐treated mice showed decreased level of TNF‐α compared with AD control group, which indicated that the MSPM administration could effectively alleviate the Aβ‐induced inflammatory in vivo. Caspase‐3 and cleaved caspase‐3 staining was performed to assess the extent of neuronal apoptosis. As shown in Figure 5d–f, although there was no obvious difference in expression of caspase‐3, the level of cleaved caspase‐3, which is the active form of caspase‐3 and more related to apoptosis, was significantly increased in APP/PS1 mice treated with saline. In contrast, mice with micelle treatments displayed lower levels of cleaved caspase‐3 and APP/MSPM group showed a more remarkable decrease. The level of caspase‐3 in APP/MSPM group was also decreased. All these data demonstrated that MSPM treatment could prominently reduce neuronal apoptosis in brain.

Furthermore, we also used hematoxylin and eosin (H&E) staining to evaluate the damage of nerve cells in hippocampus. As seen in Figure S16 (Supporting Information), compared with WT control, marked cell shrinkage and obvious nuclear pyknotic were observed in APP/saline group, indicating the occurrence of neuron damage in AD mice. With the treatment of micelles, the damages of neuronal integrity were markedly attenuated which revealed that these micelles could protect neuron efficaciously. Similarly, the neurons protective effect of MSPM was superior to that of PM. Thus, all above results indicated that our nanochaperone could effectively not only decrease amyloid deposition but also alleviate nerve injury in AD mice brain, thereby confirming the disease‐modifying effectiveness of MSPM in vivo.

## 3 Conclusion

In summary, we have successfully fabricated a biocompatible self‐assembly nanochaperone based on surface adjustable MSPM, which could effectively suppress early progression of AD. With the unique surface hydrophobic domains of bioinspired structure, this MSPM‐based nanochaperone exhibits selective Aβ‐binding affinity even under the interference of other proteins in complex biological milieu, thereby inhibiting Aβ aggregation and mitigating Aβ‐mediated cytotoxicity, as well as facilitating microglia‐mediated Aβ elimination and alleviating Aβ‐induced inflammation, thus protecting nerve cells against apoptosis. Most importantly, the experimental results in APP/PS1 transgenic mice indicated that nanochaperone administration could decrease amyloid accumulation, attenuate inflammatory and rescue cognitive deficits in the early stage of AD. Both in vitro and in vivo experiments certified the admirable efficacy of nanochaperone on reducing Aβ burden and protecting neuron, resulting in its remarkable prevention efficacy on the onset of AD. To the best of our knowledge, this is the first evidence of polymeric nanostructure that exhibits excellent in vivo effectiveness for AD treatment, which is just benefit from the own structure and character of nanochaperone without any help from peptides or antibodies. Therefore, we believe that this nanochaperone based on MSPM would be a promising prophylactic treatment strategy for early AD in clinical application.

## 4 Experimental Section

*Materials*: Aβ_42_ and FITC‐Aβ_42_ (>95%) were customized from GL Biochem Ltd. (Shanghai, China). BSA was purchased from Dingguo (Beijing, China) and ubiqintin (Ub) were purchased from Univ‐bio (Shanghai, China). ε‐Caprolactone (ε‐CL) (Alfa Aesear) was distilled under reduced pressure before utilization. Methoxy poly(ethylene glycol) (CH_3_O‐PEG_114_‐OH, *M*_n_ = 5000) and tert‐butoxycarbonyl aminoethyl poly(ethylene glycol) (BOC−NH−PEG_114_−OH; *M*_w_ = 5000) were purchased from Yarebio Ltd. (Shanghai, China) and used after dried under vacuum. Hexane‐1,6‐dioldiacrylate (HDD, 99%, Alfa Asear), 4,4'‐trimethylene dipiperidine (TDP, 97%, Alfa Asear), 2‐hydroxyethyl acrylate (HEA ,97%, J&K), stannous octoate (Sn(Oct)_2_, 96%, Alfa Asear), thioflavin T (ThT, 98%, J&K), and 1,1,1,3,3,3‐hexafluoro‐2‐propanol (HFIP, > 99%, Sigma‐Aldrich) were used as received. Cell counting kit‐8 (CCK‐8) (Dojindo, Japan), NO assay kit (Beyotime Biotechnology, China), TNF‐α ELISA kit (BD Biosciences, America), and Aβ_42_ ELISA kit (Jianglai, China) were used according to the instructions. The cell line PC‐12 and BV‐2 were purchased from cell bank of Shanghai Institutes for Biological Science (SIBS).

*Synthesis of Block Copolymers*: PEG‐*b*‐PCL and BOC‐NH‐PEG‐*b*‐PCL were synthesized through the ring opening polymerization (ROP) of ε‐CL monomer with PEG‐OH or BOC‐NH‐PEG‐OH as macroinitiators, utilizing Sn(Oct)_2_ as the catalyst. After removing the Boc group, the Cy3‐PEG‐*b*‐PCL was synthesized by acylation reaction between Cy3‐NHS and NH_2_‐PEG‐*b*‐PCL. PCL‐*b*‐PAE was synthesized through the ROP of ε‐CL and Michael‐type addition polymerization. The synthesis and characterization details were shown in the Supporting Information.

*Preparation of MSPMs*: PEG‐*b*‐PCL and PAE‐*b*‐PCL were dissolved in tetrahydrofuran (THF) first with a concentration of 5 mg mL^−1^ respectively. To afford different compositions of the MSPMs, the original solution of polymers was mixed at different ratios (1:0, 2:1, 1:1, and 1:2, v/v) first, and added dropwise into HCl solution (pH ≈ 2.0) under vigorous stirring. After bath‐sonicated for 20 min, the solution was dialyzed (molecular cut off: 3.5 KD) against PBS (pH 7.4) for 3 d to completely remove THF and MSPMs were finally obtained.

*Aβ Preparation*: Aβ_42_ was predissolved in HFIP and stored at −20 °C. Before used, the sample was evaporated with N_2_, and redissolved in anhydrous dimethyl sulfoxide (DMSO), followed by diluting to the target concentration with PBS (10 × 10^−3^ m, pH 7.4) buffer. Oligomers were obtained by storage the fresh solution at 4 °C for 24 h.

*ThT Fluorescence Assay*: For ThT fluorescence assay, the original samples were prepared by mixing Aβ monomer solution (40 × 10^−6^ m) with varied micelles (0.5 mg mL^−1^) by the volume ratio of 1:1 and incubated at 37 °C. 25 µL of each sample was withdrawn at different time intervals and mixed with 800 µL of 15 × 10^−6^ m ThT buffer solution as the test sample. The fluorescence was recorded at 485 nm with excitation wavelength of 450 nm.

*TEM*: MSPMs, Aβ, and their mixture were observed by Talos F200c electron microscope (acceleration voltage of 200 kV). The micelles were used as obtained. The protein samples were prepared as ThT assay. Aβ and its coincubated samples were incubated at 37 °C for 5 d. 10 µL of the sample was dropped onto a carbon‐coated copper grid for 10 min and blotted with filter paper to remove excess liquid. Samples contained Aβ were further negatively stained with 2% phosphotungstic acid solution, blotted again, and air‐dried before observed on TEM.

*SDS‐PAGE Assay*: The samples were prepared by mixing Aβ peptide with BSA and ubiquitin evenly and then incubating them with or without PM and MSPM respectively (final concentration: *C*(each protein) = 0.1 mg mL^−1^, *C*(micelles) = 0.25 mg mL^−1^). The mixtures were then separated by ultrafiltration for 10 min at 11 000 rpm speed (molecular cut off: 100 kD). A control experiment was performed under identical conditions that without any micelles. 100 µL PBS was added to the interception liquid and ultrafiltration again to wash the micelles and repeated for two more times. 50 µL PBS was added to the interception liquid and oscillated for 30 s to get the bound protein sample. The filtrate was used directly as the free protein sample. These samples were mixed with SDS‐containing buffer and heated at 99 °C for 3 min. The SDS‐PAGE gel was showed in silver staining protocol. The results were quantified by gray scale analysis of the gels. Three bands of the same protein in each gel (free or bound proteins gel) were defined as a group. The relative intensity is the ratio of the intensity of each band to the strongest band in its group. The only exception was the Ub‐bound group. Since there were no obvious bands of ubiquitin in bound protein gel, which indicating that the interaction between nanochaperones and ubiquitin was very weak, and we paid more attention to compare the binding ability of nanochaperones to ubiquitin and Aβ, the data of Ub‐bound group were obtained by the ratio of the intensity of bands in Ub‐bound group to the strongest bands in Aβ‐bound group.

*QCM‐D Measurement*: QCM‐D measurements were performed using a Q‐Sence E4 system (Q‐Sence, Sweden). The micelles covered chips were prepared by immersing in a solution containing TCEP and OPSS‐MSPMs (0.5 mg mL^−1^) for 24 h. Then the chip was rinsed with deionized water and dried with nitrogen gas. During the experiment, each chip was docked into the standard flow module and filled with flow buffer (PB, 10 × 10^−3^ m, pH 7.4) until the baseline was stable. The flow rate was 10 µL min^−1^. Then, different protein solutions (0.125 mg mL^−1^) in the flow buffer were injected for 30 min at a rate of 10 µL min^−1^ followed by continuous flow of the same buffer. The operating temperature of sensor was maintained at 37 °C during the experiments.

*Cell Culture*: PC‐12 and BV‐2 cells were cultured in DMEM (Gibco BRL) medium containing 10% fetal bovine serum at 37 °C in humidified 5% CO_2_. Unless explicitly noted, the final concentration of Aβ and micelles in cell experiments were: *C*(Aβ) = 2 × 10^−6^ m, *C*(micelles) = 0.25 mg mL^−1^.

*Cell Toxicity Assay*: For the CCK‐8 assay, cells were plated at a density of 5000 cells per well on 96‐well plates overnight. After incubation of 24 h, materials were added to the cells. (Final concentration: *C*(Aβ oligomer) = 10 × 10^−6^ m, *C*(micelles) = 25, 50, 100, 200, 400 µg mL^−1^) 24 h later, 100 µL of tenfold diluted CCK‐8 solution was used to replace the mixture in each well and the cells were further incubated for another 4 h. Absorbance values were measured at 465 nm using a NanoDrop Onec (Thermo Scientific, USA). Cells without micelles were used as control.

*Cellular Adherence*: For confocal laser‐scanning microscopy, 10^4^ PC‐12 cells were plated on confocal dishes overnight in fresh medium. After incubation at 37 °C of 24 h, FITC‐Aβ and micelles were added into the cells in sequence. After the further incubation for 2 h, the cells were washed three times with PBS buffer. Then the cells were fixed with 4% polyformaldehyde solution, and the nuclei were stained with DAPI. 0.5 mL PBS was added for observation. For flow cytometry, 4 × 10^4^ cells were plated in each well on 12‐well plates in fresh medium. The cells were treated as the above method. After the incubation, cells were washed three times with PBS buffer and detached by 0.02% (w/v) EDTA and 0.25% (w/v) trypsin solution. After centrifugation, cells were dispersed in 0.5 mL of 4% polyformaldehyde solution for flow cytometric measurement. Cells treated with PBS were used as control.

*Cellular Phagocytosis of Aβ*: For confocal laser‐scanning microscopy, the BV‐2 cells were plated as above. After incubation at 37 °C of 24 h, the medium was changed to serum‐free DMEM to culture for 1 h before the addition of micelles. FITC‐Aβ and micelles were premixed for 15 min, and then added to the cells. After incubation for 2 h, the cells were washed three times with PBS buffer. Then the cells were fixed with 4% polyformaldehyde solution and 0.5 mL PBS was added for observation. The lyso‐tracker red dye was added 1 h before the PBS wash. For flow cytometry, cells were plated on 12‐well plates, and treated as the above method. After the incubation, cells were handled as same as PC‐12 cells.

*Cell Uptake of Nanochaperone and Nanochaperone‐Aβ Complex*: The Cy3 labeled micelles were prepared utilizing Cy3‐PEG‐*b*‐PCL and the content of Cy3‐PEG‐*b*‐PCL in both PM and MSPM were ensured to be the same. The fluorescence intensity of both micelles with the same concentration was recorded at 570 nm with excitation wavelength of 550 nm. BV‐2 cells were separately seeded into 12‐well plates with a density of 4 × 10^4^ cells per well. The micelle‐Aβ complexes were prepared by incubating the Cy3 labeled micelles with Aβ for 15 min. The Cy3 labeled micelles and the complexes were added to the cells and incubated at 37 °C of 2 h, then the cells were treated as above for confocal laser‐scanning microscopy and flow cytometric measurement.

*Measurement of TNF‐α and Nitrite Levels*: BV‐2 cells were plated on 12‐well plates in fresh medium. After incubation at 37 °C of 24 h, the medium was changed to serum‐free DMEM and cells were stimulated with Aβ oligomers (final concentration: 10 µM) and micelles for 24 h. The supernatants were collected for assays of the levels of TNF‐α and nitrite. The TNF‐α level was measured by mouse TNF‐α ELISA kit (BD, America). As the indicator of NO production, the level of nitrite was detected through the Griess assay by NO detection kit (Beyotime, China).

*Animals*: 5 months old adult male Balb/C, C57BL/6J, and APPswe/PS1ΔE9 mice were obtained from the Institute of Zoology, Chinese Academy of Science. All experimental protocols were approved by the Local Animal Care Committee at Nankai University (State Key Laboratory of Medicinal Chemical Biology of Nankai University) and all methods were performed in accordance with this guideline and regulation.

*Intracerebral Injection*: Mice were processed surgery at 5 months old. C57 and APP/PS1 mice were randomly divided into three groups respectively to receive saline, PM and MSPM. The mice were anesthetized and fixed in a stereotaxic apparatus before surgery. The micelle concentration was concentrated to 4 mg mL^−1^ by rotary evaporation. The used materials were injected into CA1 area of unilateral hippocampus (−2.0 mm anteroposterior, ±1.3 mm mediolateral, −1.3 mm dorsoventral) with the current speed at 2 µL min^−1^ for 2 min. Mice were injected with saline for the sham surgery and as control groups.

*Study of the Retention and Distribution of Nanochaperones in Mouse Brain*: TPETPAFN was encapsulated in MSPM as a near‐infrared fluorescence probe to indicate the presence of micelles in mice brain and Balb/C mice were used as the animal model. The mice were anesthetized and visualized under in vivo fluorescence imaging system at different time. To further investigate the retention and distribution of nanochaperones in the brain, C57 mice were used as the model mice and sacrificed at different time after the intracerebral injection of Cy3 labeled MSPM. Brains were removed and made into sections (2 mm) for fluorescence imaging or frozen sections (10 µm thick, DAPI staining) for confocal laser‐scanning microscopy.

*Quantification of Nanochaperones in Bilateral Cerebral Cortex and Hippocampus*: C57 mice were intracerebrally injected with Cy3 labeled MSPM and sacrificed at different time. The bilateral cerebral cortex and hippocampus were isolated from mouse brain and separately homogenized with PBS buffer. After centrifugation, 50 µL of each original sample was mixed with 800 µL PBS as the test sample. The fluorescence was recorded at 650 nm with excitation wavelength of 550 nm.

*Object Recognition Task*: The ORT was performed according to literature description.^47^ The apparatus and objects (cubes and tetrahedrons) were cleaned with 75% ethanol between subjects to eliminate odor cues. 24 h before the test, the mice were allowed 30 min to familiarize themselves with the arena. In the sample phase trial, each mouse was placed into the apparatus to exposure to two identical objects for 5 min. 24 h later, one of the objects was replaced by the novel one and the mice were placed into the apparatus to explore for another 5 min. Their behaviors were recorded by a video tracking system and the exploring time on each object was recorded. The time spent on exploring the different objects was calculated to measure the memory performance of mice.

*Morris Water Maze Task*: The spatial cognitive function was tested by Morris Water Maze Task (MWM). The maze included a circular swimming pool filled with 25 °C water and a small escape platform. The water was opaque with nontoxic biodegradable lime dye to hide the installed platform from the sight of mice. Mice can remember the location of the platform based on special cues on the surrounding walls. During each of the four trials per day, experimental mice were placed into the pool in four different quadrants of the circle along the edge. Each trial lasted until the animal found the platform or for a maximum of 120 s. If a mouse failed to find the platform, it would be guided to the platform and remained for 30 s. The escape latencies and distances that the mice toke to find the hidden platform were recorded to measure the spatial cognitive function of mice.

*Immunofluorescence Staining*: Animals were euthanized after the behavior assessment. The brain tissues were harvested and fixed in 10% formalin, embedded in paraffin, and cut into sections (3 µm thick). After the antigen retrieval, the sections were blocked by goat serum for 30 min. A battery of primary antibodies, including rabbit anti‐Aβ (1:200, Abcam, ab201060), rat anti‐IBA‐1 (1:200, Abcam), rabbit anticaspase 3 (1:200, Servicebio), rabbit anticleaved caspase 3 (1:200, CST), were diluted in blocking solution and incubated with sections overnight at 4 °C. After incubation, the sections were washed by PBS and then incubated with secondary antibodies of goat antirabbit (Alexa 488, Abcam) or goat antirat (Alexa 594, Abcam). DAPI Fluoromount‐G (Southern Biotech) was used for the nuclear counterstaining. To investigate the colocalyzation of nanochaperones with microglia, Cy3 labeled MSPM were injected in to APP/PS1 mice and the mice were sacrificed after 48 h. The brain tissues were made into a frozen section (10 µm thick), following incubating with the primary antibody, rabbit anti‐IBA‐1 (1:200, Abcam), and the secondary antibody, goat antirabbit (Alexa 488, Abcam) in sequence.

*Western Blot*: The whole brain of mouse was immediately lysed in a tissue protein extraction reagent (CWBIO, China) with PMSF (Sigma‐Aldrich) after the harvest. Protein concentrations were first quantified using BCA Protein Assay Kit (CWBIO). Samples containing equal amount of proteins were loaded on SDS‐PAGE gels and transferred to a nitrocellulose membrane. After being blocked, the membrane was incubated with primary antibodies, including rabbit anti‐Aβ (1:1000, Abcam, ab201060), rabbit anti‐TNF‐α (1:1000, Abclonal, A0277), rabbit anticaspase 3 (1:1000, Servicebio), rabbit anticleaved caspase 3 (1:1000, CST) and mouse antiactin (1:3000, Servicebio) overnight at 4 °C. Then the membrane was incubated with appropriate horseradish peroxidase‐conjugated secondary antibody (1:3000, CWBIO), subsequently detected by SuperSignal West Pico chemiluminescent substrate (Thermo Scientific). The band density was all normalized to β‐actin when analyzing.

*Quantification of Aβ in Bilateral Cerebral Cortex and Hippocampus*: The bilateral cerebral cortex and hippocampus were isolated from mouse brain and separately homogenized using guanidine buffer with proteinase inhibitor cocktail. After centrifugation, the Aβ_42_ levels in the homogenate were measured by ELISA assay.

*Hematoxylin/eosin Staining*: Formalin‐fixed, paraffin‐embedded brain sections were dewaxed and rehydrate and were stained by H&E in sequence. The hippocampal areas were examined under a Leica optical microscope (Leica, Germany).

*Statistical Analysis*: All data were presented as the mean ± standard deviation (S.D.). Statistical analysis of the data was performed using One or Two‐way ANOVA or Student's *t*‐tests method and *p*‐value of <0.05 was considered significant.

## Conflict of Interest

The authors declare no conflict of interest.

## Supporting information

Supplementary
