# Supplementary material for: Heat Shock Protein Inspired Nanochaperones Restore Amyloid‐β Homeostasis for Preventative Therapy of Alzheimer's Disease
Source: Adv Sci (Weinh). 2019 Sep 16;6(22):1901844. doi: 10.1002/advs.201901844 (PMC6864524; doi:10.1002/advs.201901844)
Supplement: Supplementary file 1 — Supplementary [file ADVS-6-1901844-s001.pdf]

Copyright WILEY-VCH Verlag GmbH & Co. KGaA, 69469 Weinheim, Germany, 2019.

ADVANCED SCIENCE
Open Access

Supporting Information

for *Adv. Sci.,* DOI: 10.1002/advs.201901844

Heat Shock Protein Inspired Nanochaperones Restore Amyloid-# Homeostasis for Preventative Therapy of Alzheimer’s Disease

Huiru Yang, Xinyu Li, Lin Zhu, Xiaohui Wu, Shaozhi Zhang, Fan Huang, Xizeng Feng,* and Linqi Shi**

Supporting Information

Heat Shock Protein Inspired Nanochaperones Restore Amyloid-β Homeostasis for Preventative Therapy of Alzheimer's Disease

Huiru Yang, Xinyu Li, Lin Zhu, Xiaohui Wu, Shaozhi Zhang, Fan Huang, Xizeng Feng* and Linqi Shi**

H. Yang, L. Zhu, X. Wu, Prof. L. Shi,

State Key Laboratory of Medicinal Chemical Biology, Key Laboratory of Functional Polymer Materials, Ministry of Education, Institute of Polymer Chemistry, College of Chemistry, Nankai University Department

Tianjin 300071, P. R. China

E-mail: shilinqi@nankai.edu.cn

Dr. X. Li, S. Zhang, Prof. X. Feng,

State Key Laboratory of Medicinal Chemical Biology, Key Laboratory of Bioactive Materials, Ministry of Education, College of Life Sciences, Nankai University,

Tianjin, 300071, P. R. China

E-mail: xzfeng@nankai.edu.cn

Dr. F. Huang

Tianjin Key Laboratory of Radiation Medicine and Molecular Nuclear Medicine, Institute of Radiation Medicine, Chinese Academy of Medical Sciences & Peking Union Medical College

Tianjin 300192, P. R. China

E-mail: huangfan@irm-cams.ac.cn

Synthesis of Block Copolymers

Synthesis of PEG-*b*-PCL

PEG-*b*-PCL was synthesized through the ring opening polymerization (ROP) of ε-CL monomer with PEG-OH as the macro-initiators utilizing $Sn(Oct)_2$ as the catalyst. CH_3O-PEG-OH, ε-CL monomer and one drop of $Sn(Oct)_2$ were dissolved in 10 ml of toluene. After freeze-degas-thaw cycles for three times, the reaction mixture was stirred at 110 °C for 12 h. Then the solution was precipitated with excess diethyl ether and filtrated to obtain the product.

Synthesis of Cy3-PEG-*b*-PCL

BOC-NH-PEG-*b*-PCL was synthesized in the same way as PEG-*b*-PCL by replacing the macro-initiators PEG-OH with BOC-NH-PEG-OH. In order to obtain NH_2-PEG-PCL, the BOC-NH-PEG-*b*-PCL was dissolved in 10 mL CH_2Cl_2 with excessive trifluoroacetic acid, and stirred for 24 h at room temperature. The mixture was precipitated with excess diethyl ether and filtrated. After removing the Boc group, the Cy3-PEG-*b*-PCL was synthesized by acylation reaction between hydrophilic dye Cy3 and NH_2-PEG-b-PCL. Briefly, Cy3 NHS ester, NH_2-PEG-PCL and trimethylamine were dissolved in DMF and then stirred in the dark for 24 h at room temperature. The solution was then dialyzed (molecular cut off: 3.5 KD) against water for 3 days to completely remove DMF. Cy3-PEG-*b*-PCL was eventually obtained by lyophilization.

Synthesis of PAE-*b*-PCL

PCL-*b*-PAE was synthesized through the ROP of ε-CL and Michael-type addition polymerization. ε-CL, 2-hydroxyethyl acrylate and one drop of $Sn(Oct)_2$ were dissolved in 10 mL of toluene. The reaction was same with the synthesis of PEG-*b*-PCL and the product PCL-A with vinylic groups in one end was obtained. Then, PCL-A, HDD and TDP were dissolved in 10 mL of $CHCl_3$. After stirred for 72 h at 55 °C, the solution was precipitated into excess diethyl ether to obtain PCL-*b*-PAE. All the products were dried under vacuum.

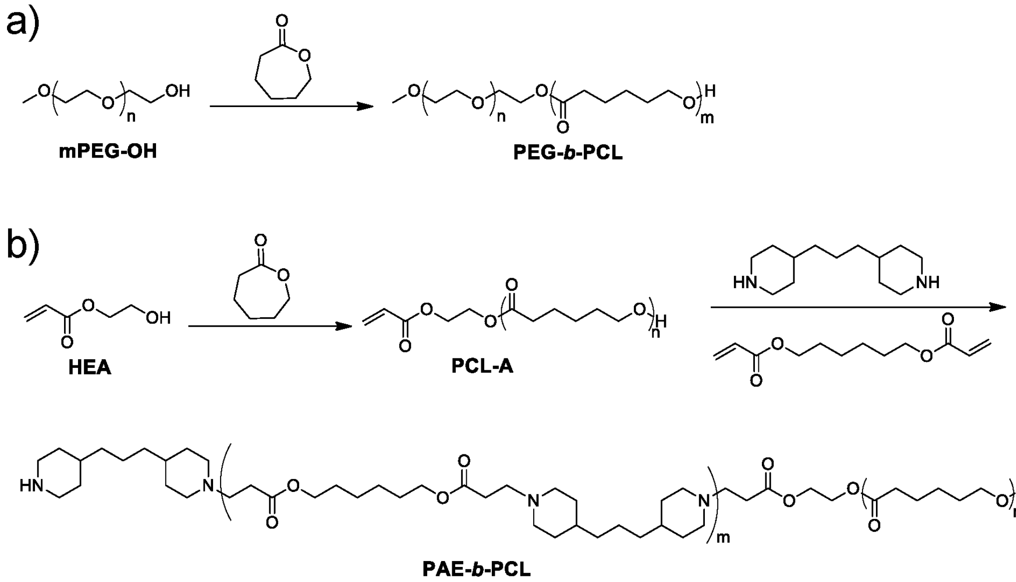

Figure S1. Synthesis routes of PEG-*b*-PCL (a) and PAE-*b*-PCL (b).

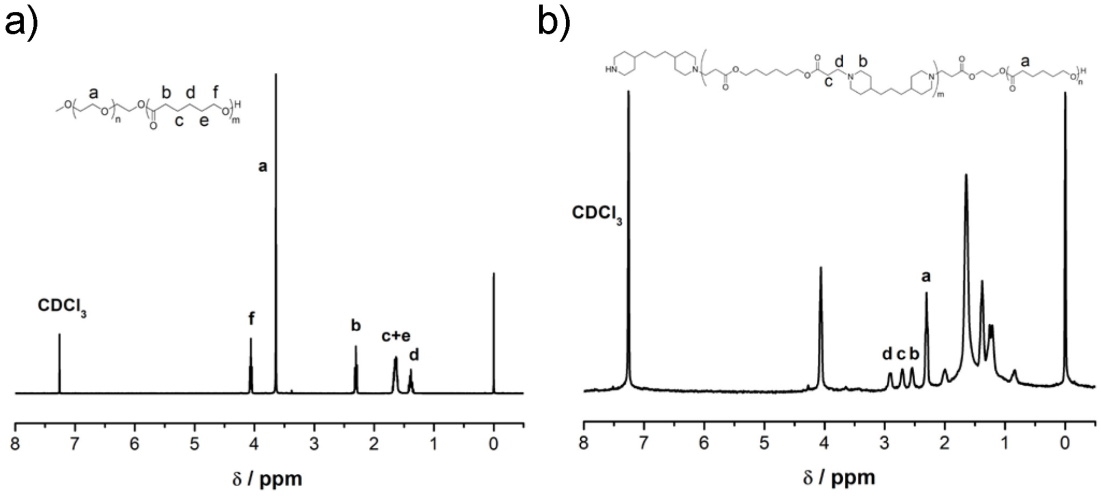

Figure S2. ^{1}H NMR results of PEG-*b*-PCL (a) and PAE-*b*-PCL (b).

a)

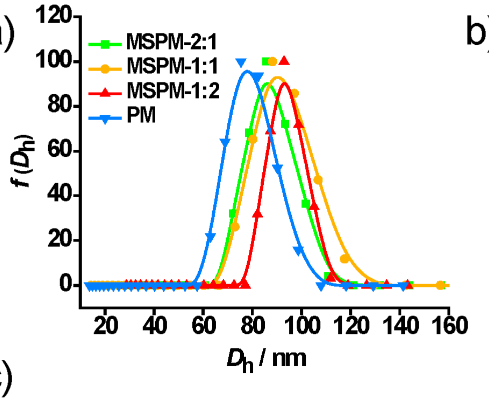

b)

Code	PEG:PAE (w/w)	D_h (nm)
PM	1:0	75.36
MSPM-2:1	2:1	85.70
MSPM-1:1	1:1	88.26
MSPM-1:2	1:2	92.92

c)

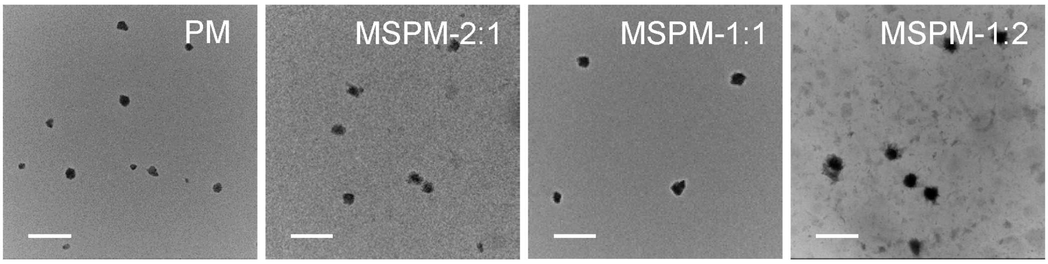

Figure S3. Hydrodynamic diameter distribution (a), average values (b) and TEM images (c) of the PM and MSPMs with different weight ratios. Scale bar = 200 nm.

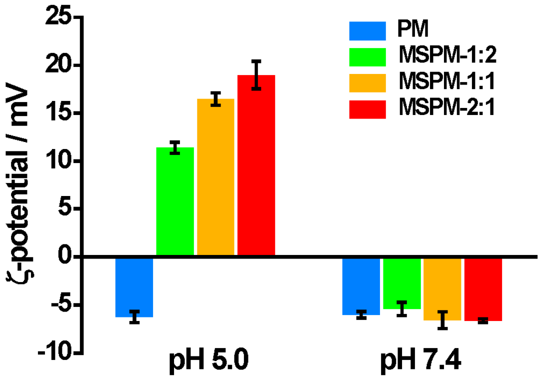

Figure S4. Zeta potential of the PM and MSPMs at different pH.

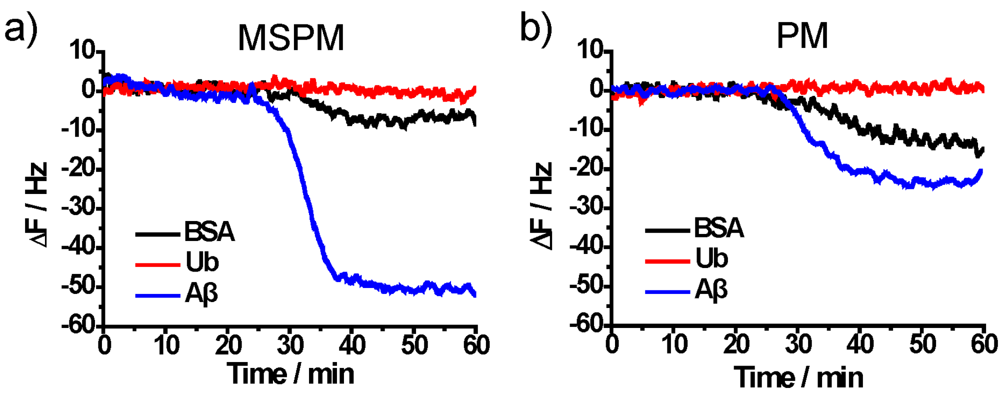

Figure S5. QCM-D analysis of binding affinity of MSPM (1:1, w/w) (a) and PM (b) to different proteins.

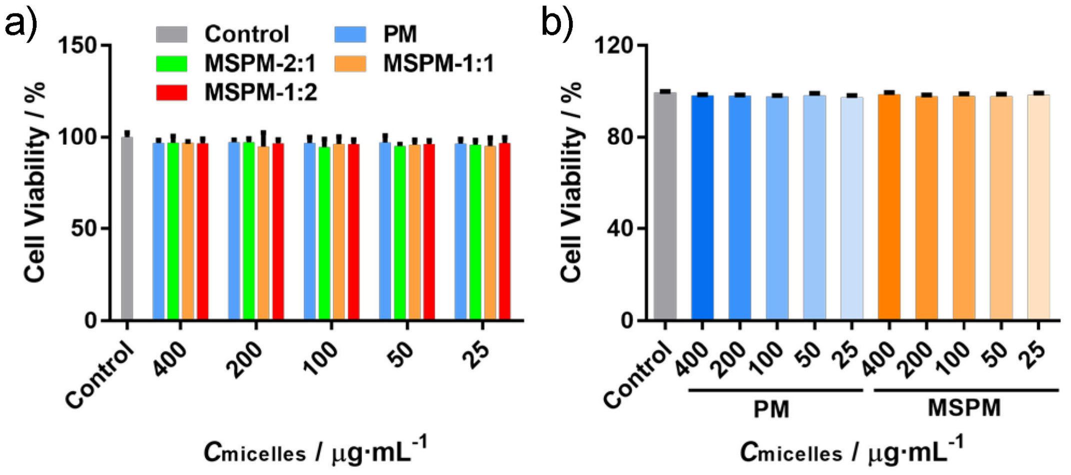

Figure S6. Cytotoxicity of nanochaperones against PC-12 cells (a) and BV-2 cells (b). The concentration of micelles was 0 (control), 25, 50, 100, 200, 400 μg/ml, respectively. For BV-2, MSPM means MSPM-1:1. Cell viability was measured by CCK-8 assay. Data were presented as mean ± SD, n = 3.

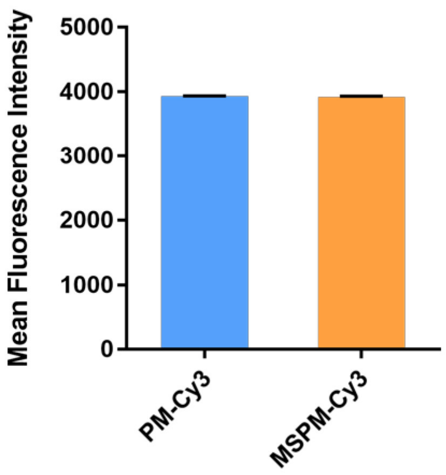

Figure S7. Fluorescence intensity of PM and MSPM with the same concentration of 0.5 mg/mL. Data were presented as mean ± SD, n = 3.

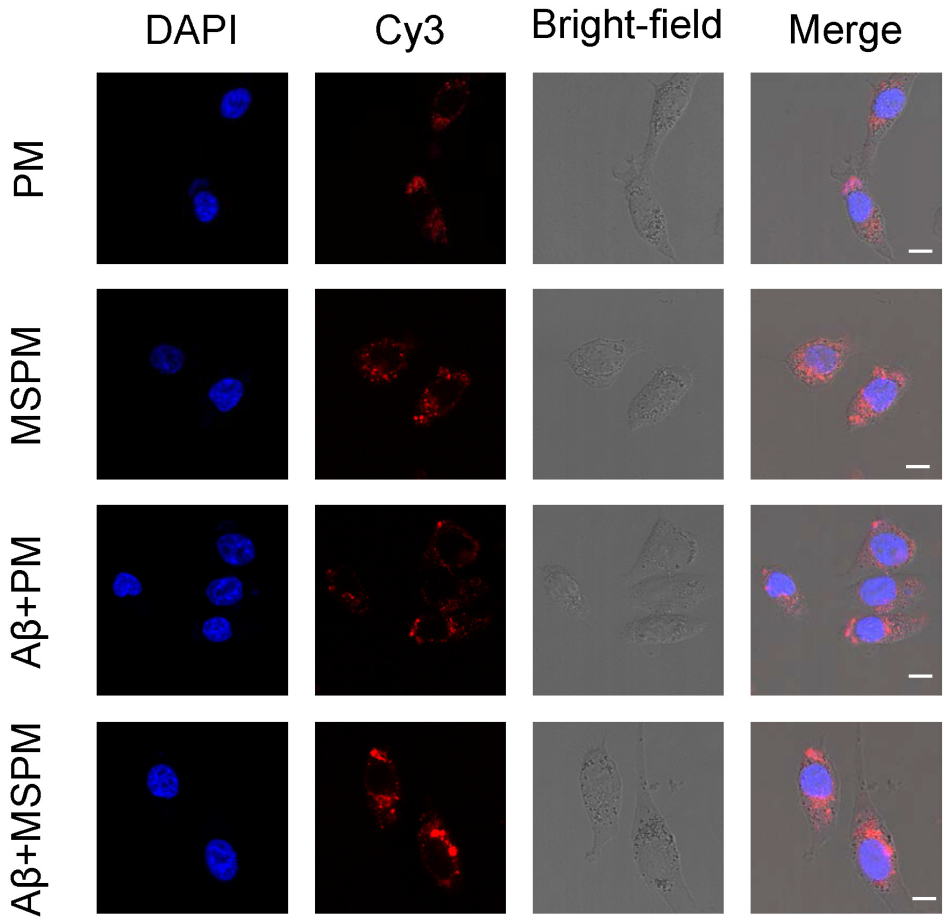

Figure S8. CLSM micrographs of BV-2 cells after incubation with nanochaperones or nanochaperone-Aβ complexes. Blue: DAPI; Red: Cy3-micelles. Bar = 10 μm.

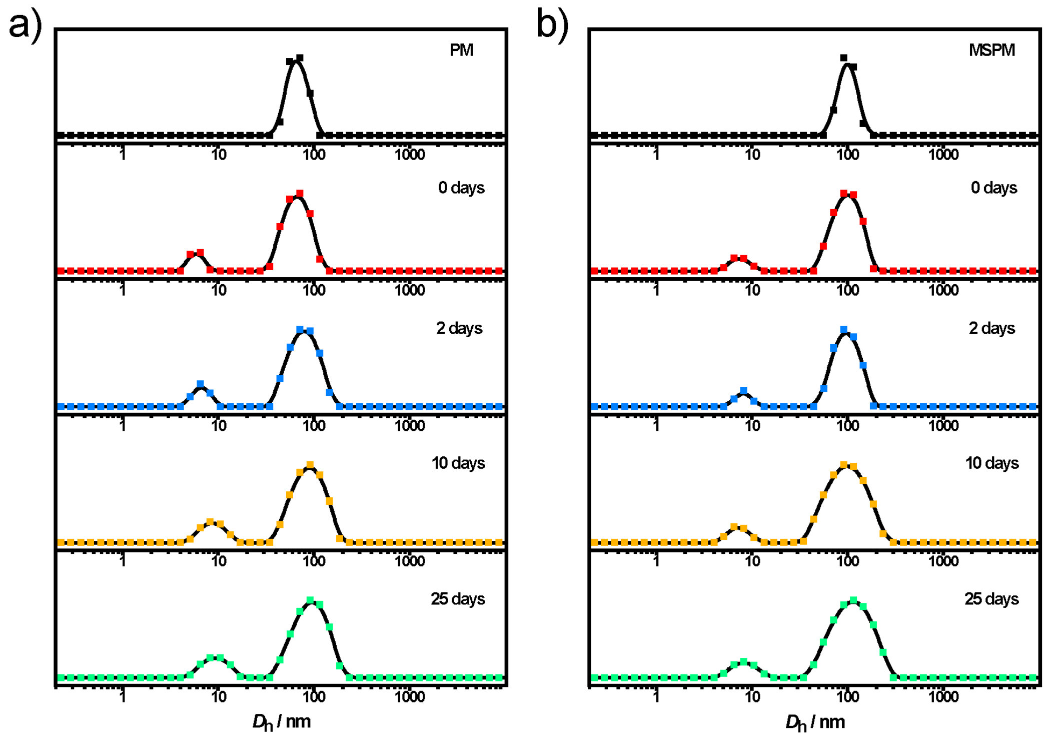

Figure S9. Size distributions of PM (a) and MSPM (b) in FBS at 37 °C. The first lines were the hydrodynamic diameter distribution of micelles without FBS at 0 days.

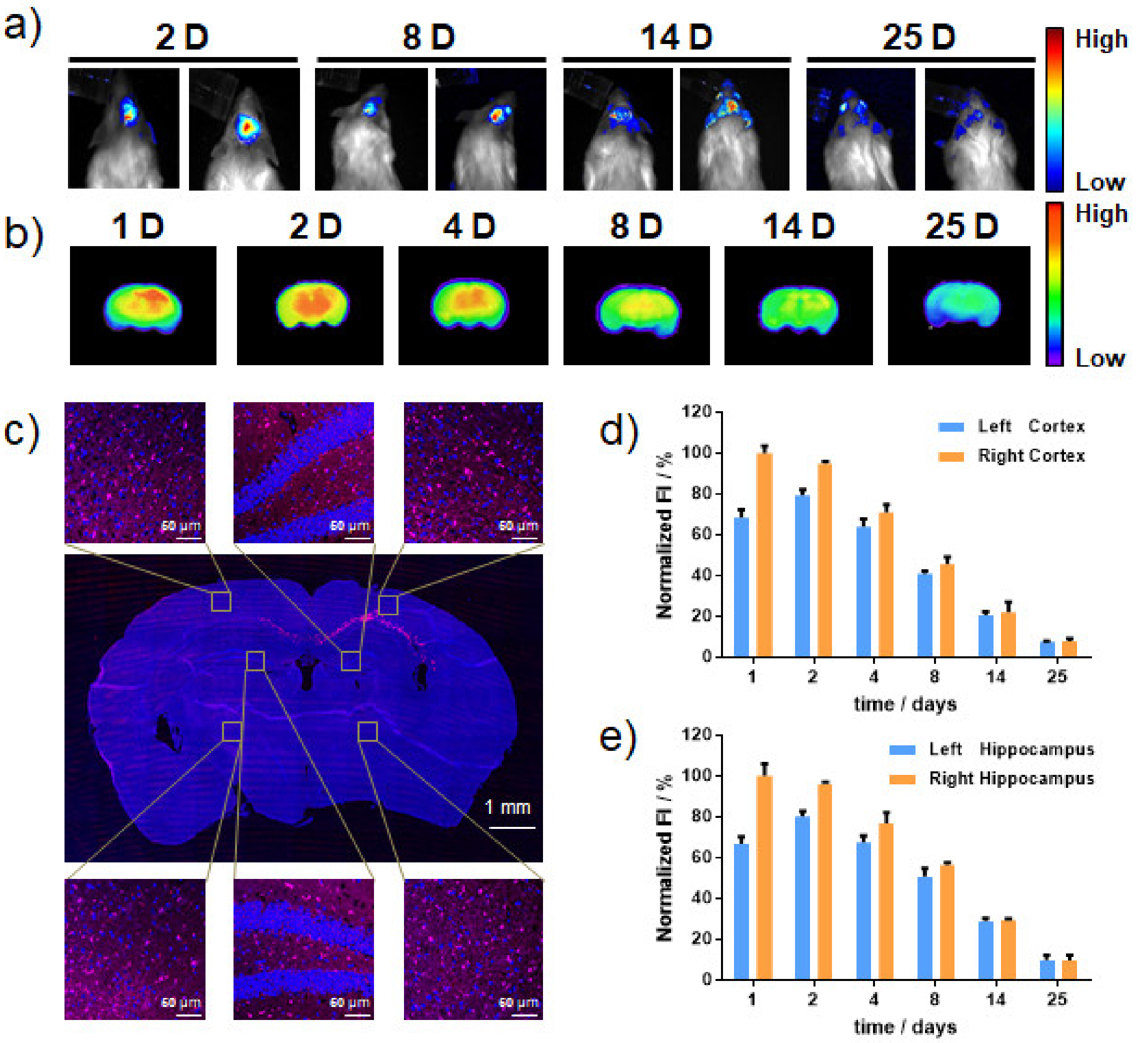

Figure S10. The retention and distribution of nanochaperones in vivo. a-b) The fluorescence imaging of mice (NIR, a) or mice brain sections (Cy3, b) after the intracerebral injection in different time. c) Typical CLSM micrographs of the distribution of MSPM in mice brains at 48 h. Blue: DAPI; Red: Cy3-MSPM. d-e) Quantitative analysis of the distribution of MSPM in different time. Data were presented as mean ± SD, n = 3. Normalized FI (Normalized Fluorescence Intensity). d) cortex; e) hippocampus.

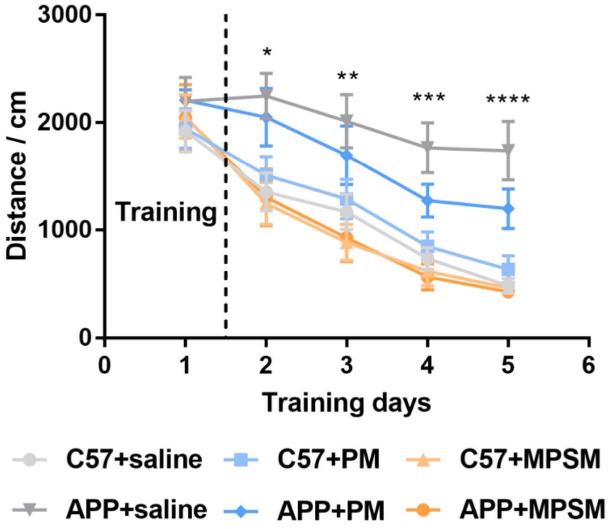

Figure S11. Swimming distance of C57 and APP/PS1 mice with saline, PM and MSPM treatment to find the hidden platform in MWM task. Data were presented as mean ± SD, (4 mice in APP/saline group and 6 mice in each other groups), Two-way ANOVA. Symbols (*) mark statistically significant difference between APP/saline group and APP/MSPM group. *P < 0.05, **P < 0.01, ***P < 0.001 and ****P < 0.0001.

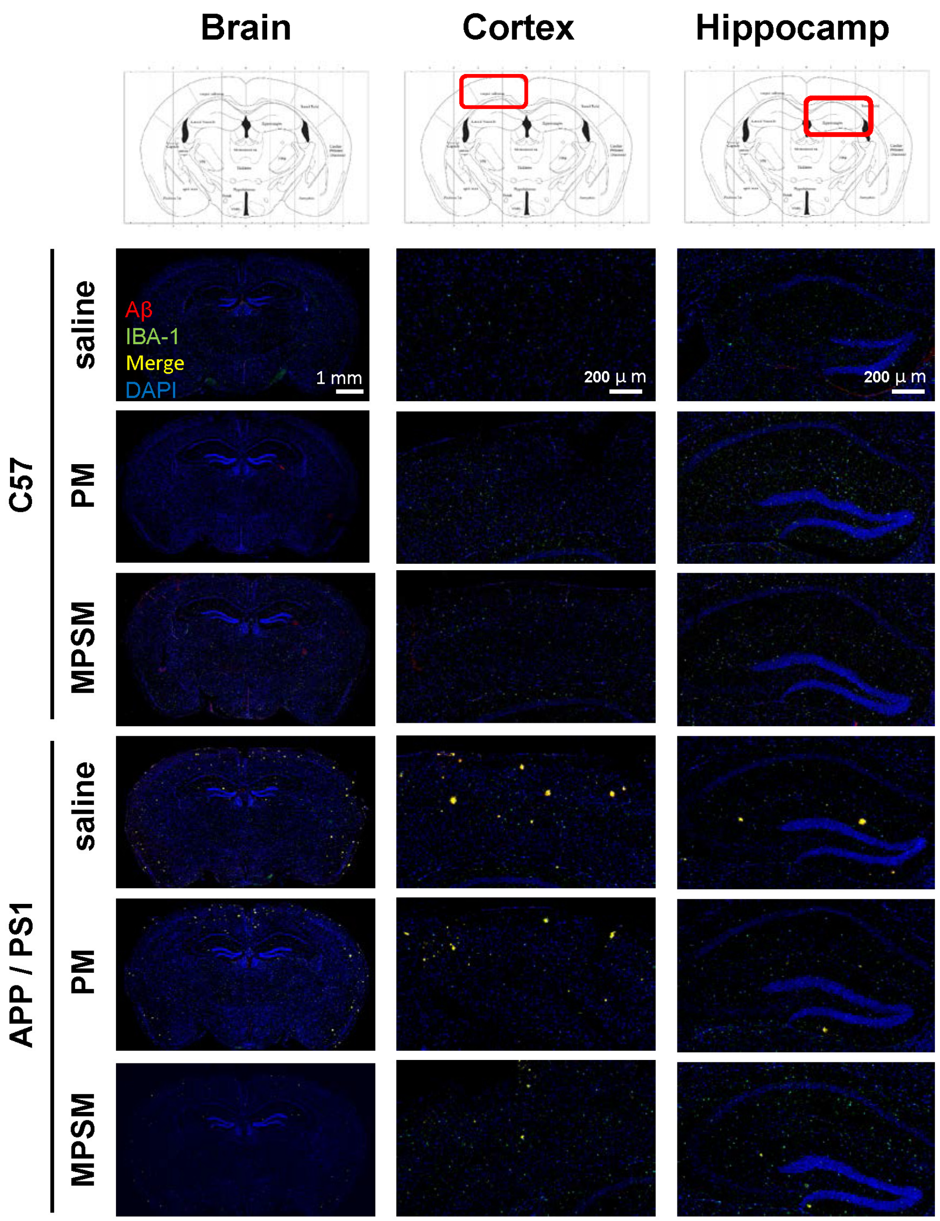

Figure S12. Immunofluorescence images for Aβ and IBA-1 expression. Aβ (red), IBA-1 (green) and merge (yellow). Whole brain, hippocampus and cortex were shown.

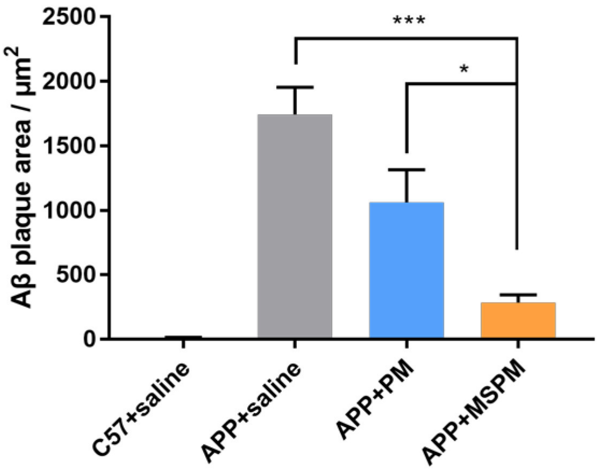

Figure S13. The average amyloid plaque sizes in mice brain. Data were presented as mean ± SD, n = 5, One-way ANOVA, Tukey's multiple comparisons test, *P <0.05, ***P < 0.001.

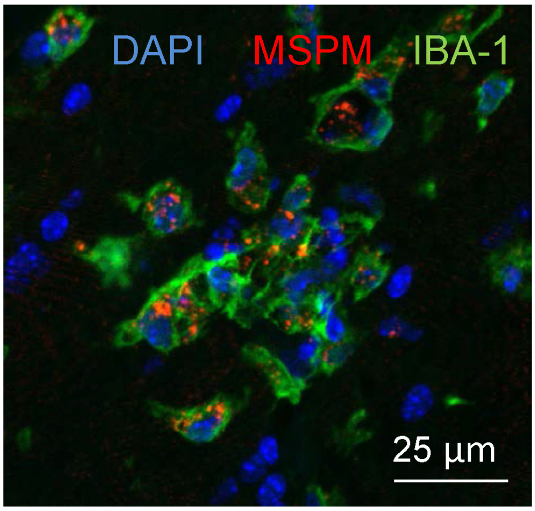

Figure S14. Immunofluorescence images for co-localization of MSPM (red) and IBA-1 (green) in the brain of APP/PS1 mice.

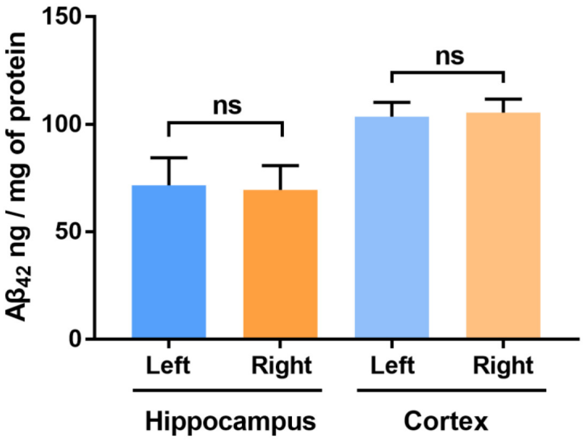

Figure S15. $A\beta_{42}$ levels in bilateral cerebral cortex and hippocampus of MSPM treated APP/PS1 mice measured by ELISA assay. Data were presented as mean ± SD, n = 4, Student's t-tests.

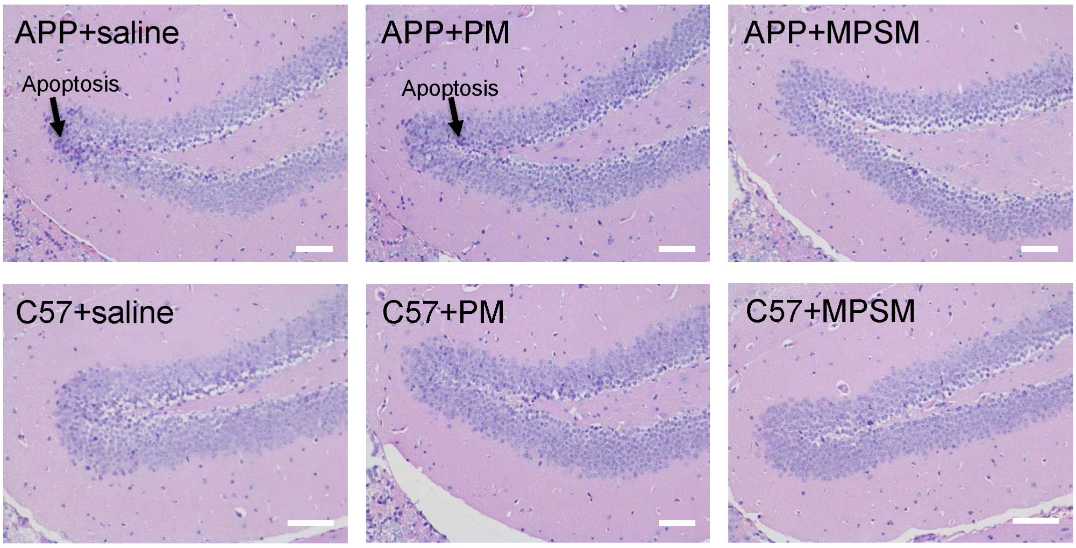

Figure S16. H&E staining of hippocampus in the brains of WT and AD mice treated with saline, PM and MSPM. Scale bar = 50 μm.
